# Effect of Low-Frequency AC Magnetic Susceptibility and Magnetic Properties of CoFeB/MgO/CoFeB Magnetic Tunnel Junctions

**DOI:** 10.3390/nano4010046

**Published:** 2014-01-02

**Authors:** Yuan-Tsung Chen, Sung-Hao Lin, Tzer-Shin Sheu

**Affiliations:** Department of Materials Science and Engineering, I-Shou University, Kaohsiung 840, Taiwan; E-Mails: isu10107009m@cloud.isu.edu.tw (S.H.L.); sheu415@isu.edu.tw (T.S.S.)

**Keywords:** magnetic tunnel junctions (MTJs), exchange coupling, low-frequency alternate-current (AC) magnetic susceptibility (χ_ac_), resonance frequency (*f*_res_)

## Abstract

In this investigation, the low-frequency alternate-current (AC) magnetic susceptibility (χ_ac_) and hysteresis loop of various MgO thickness in CoFeB/MgO/CoFeB magnetic tunneling junction (MTJ) determined coercivity (*H*_c_) and magnetization (*M*_s_) and correlated that with χ_ac_ maxima. The multilayer films were sputtered onto glass substrates and the thickness of intermediate barrier MgO layer was varied from 6 to 15 Å. An experiment was also performed to examine the variation of the highest χ_ac_ and maximum phase angle (θ_max_) at the optimal resonance frequency (*f*_res_), at which the spin sensitivity is maximal. The results reveal that χ_ac_ falls as the frequency increases due to the relationship between magnetization and thickness of the barrier layer. The maximum χ_ac_ is at 10 Hz that is related to the maximal spin sensitivity and that this corresponds to a MgO layer of 11 Å. This result also suggests that the spin sensitivity is related to both highest χ_ac_ and maximum phase angle. The corresponding maximum of χ_ac_ is related to high exchange coupling. High coercivity and saturation magnetization contribute to high exchange-coupling χ_ac_ strength.

## 1. Introduction

Since 1995, the tunneling magnetoresistance (TMR) effect has been extensively discussed, and it has been exploited in much of our modern technology [1,2]. In the past, increasing attention has been paid to ferromagnetic exchange coupling in magnetic fields [3,4], and the discovery of spintronics has led to a rapid increase in the number of exchange coupling issues. A magnetic tunneling junction (MTJ) has a trilayer structure that comprises a top free ferromagnetic (FM1) layer, an insulating tunneling barrier layer (spacer), and a bottom pinned ferromagnetic (FM2) layer. It has a great potential for use in magnetoresistance random access memory (MRAM). It provides many advantages, such as low loss energy, lack of volatility and semi-permanence features, and can be used in high-density magnetic read heads [5,6,7]. The first demonstrated MgO based tunnel junctions are Parkin *et al.* [8] and Yuasa *et al.* [9]. The mechanism of TMR in MgO based junctions is explained by Butler *et al.* [10]. In the past, TMR based on CoFeB/MgO/CoFeB MTJ has attracted considerable attention. For example, a previous study found that the magnetron sputtering of CoFeB/MgO/CoFeB at room temperature (RT) yielded a high TMR ratio [11,12]. Lee *et al.* [13] also achieved a TMR ratio of 500% at RT. Furthermore, the fabrication of high-quality junctions requires a superior ferromagnetic layer with a high spin polarization, a crystalline ordering, and a sufficient indirect spin exchange-coupling between the FM1 and FM2 layers [14,15,16,17]. The defects in the tunnel barrier material can lead to electron trapping and resistance fluctuations and induce field-dependent 1/f noise [18]. The origin of 1/f power spectrum is attributed to charge traps occurring in the barrier layer or near the interfaces between barrier and magnetic layers at low frequencies [18,19,20,21,22,23]. The alternate-current (AC) susceptibility is related to magnetic noise and exchange-coupling interaction. The high AC susceptibility can enhance a strong dipole-dipole interaction effect [24]. Moreover, a proper exchange-coupling interaction can induce a large signal-to-noise ratio [25]. However, the external stress acting on magnetic element can induce magnetic susceptibility variation of ferromagnetic layers and disturb spectral power noise of read head device. At low frequencies, the spectral power noise is dependent on free and fixed ferromagnetic layers of hysteresis loop owing to thermal magnetization fluctuations. The origin of magnetic fluctuations is excited hopping of magnetic domain walls. However, most of MTJ research has focused on the TMR, whereas the relative low-frequency alternate-current (AC) magnetic susceptibility (χ_ac_) has rarely been examined. The low field AC measurement at low frequencies is related to the spin sensitivity of MTJ devices [18]. The low-frequency AC magnetic susceptibility (χ_ac_) and hysteresis loop of CoFeB/MgO/CoFeB are worthwhile to study. This investigation focuses on the maximum χ_ac_, the optimal resonance frequency (*f*_res_) and maximum phase angle (θ_max_) for various MgO barrier thicknesses (6, 8, 11, 13, and 15 Å). The maximum χ_ac_ is 0.7 at the optimal resonant frequency of 10 Hz and the maximum phase angle is 228° at an MgO thickness of 11 Å. These values are larger than compared for Fe_40_Pd_40_B_20_(*X* Å)/ZnO(500 Å) and suitable for low-frequency magnetic media applications [26]. The magnetic material under the external AC magnetic field shows a magnetic property called multiple-frequency AC magnetic susceptibility χ_ac_ [27]. The origin of χ_ac_ is due to the association between magnetic spin interactions [27]. The frequency of the applied AC magnetic field equals the frequency of oscillation of the magnetic dipole. The maximum χ_ac_ value is corresponding to optimal resonance frequency, increasing spin sensitivity at optimal *f*_res_. It means that the optimal *f*_res_ is associated with maximal spin sensitivity.

## 2. Results and Discussion

**Figure 1:**
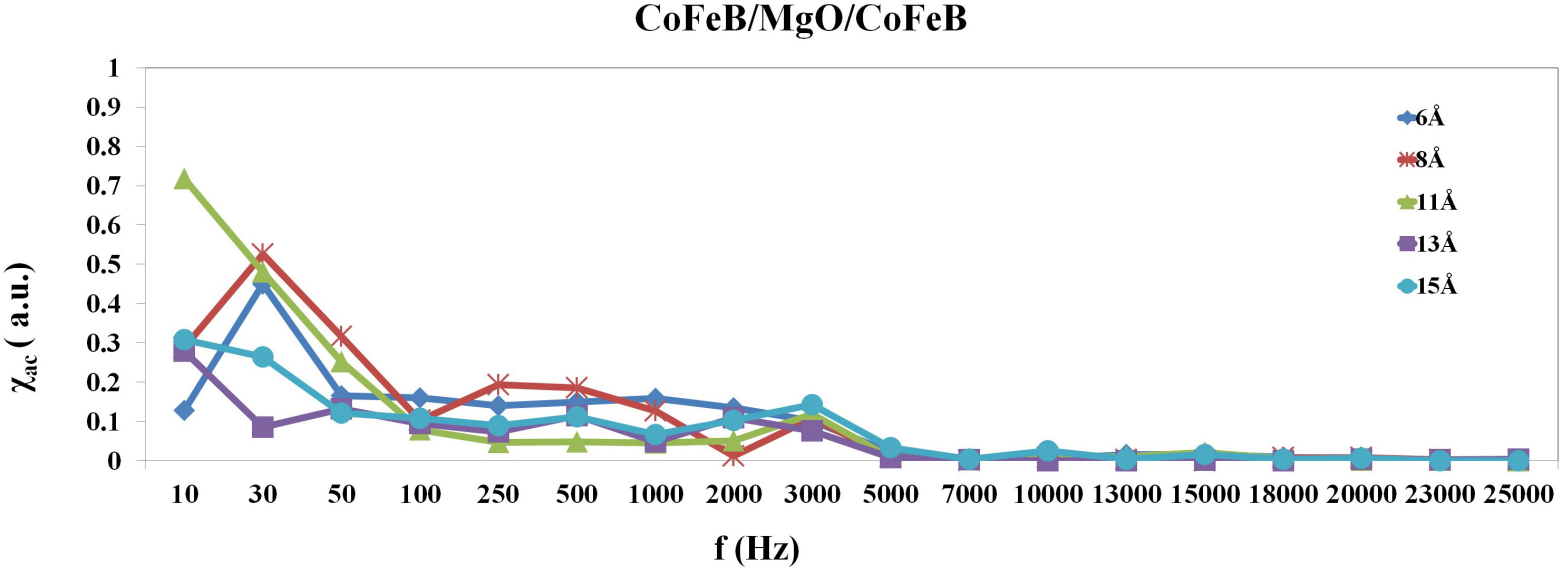
Measured low-frequency alternate-current magnetic susceptibility (χ_ac_) of CoFeB/MgO/CoFeB as a function of thickness of MgO barrier layer.

Figure 1 presents the χ_ac_ amplitude of the CoFeB/MgO/CoFeB MTJ for different thicknesses of the MgO layer at frequencies in the range 10 to 25,000 Hz. The lowest measured frequency is 10 Hz and the smallest step frequency is 20 Hz at low frequencies for used χ_ac_ measurement. The maxima χ_ac_ at the optimal resonance frequency has the following physical meaning. At low frequencies, the resultant alternate-current (AC) magnetic dipole moment is contributed from the oscillation of volume magnetic dipole moment inside each domain. The applied AC magnetic field acts a driving force. The magnetic interactions among domains act restored. There exists a resonant frequency as a driving force acting to the system. Thus, the frequency of the peak of the low-frequency magnetic susceptibility corresponds to the resonant frequency of the oscillation of the magnetic dipole moment inside domains. The χ_ac_ peak indicates the spin exchange-coupling interaction and dipole moment of domain under frequency [27]. It is reasonably concluded that the physical meaning peaks of the low frequency susceptibility indicate the magnetic exchange coupling between two CoFeB layers. The results suggest that an excitation frequency of 10 to 30 Hz maximizes the χ_ac_ of the magnetic exchange-coupled signal, and as the frequency increases above 30 Hz, the χ_ac_ obtained from the signal declines, suggesting that the CoFeB/MgO/CoFeB MTJ is suited to use at low frequencies. The optimal maximum susceptibility, at frequencies in the range of 10 to 30 Hz, can be utilized in inductors and transformers [28,29].

**Figure 2:**
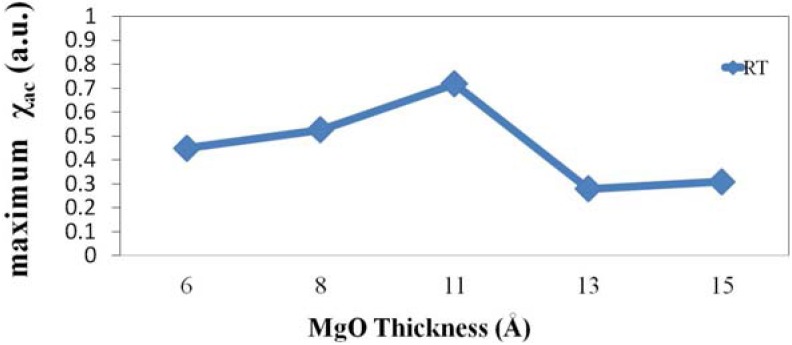
Maximum χ_ac_ as a function of thickness of MgO barrier layer.

Briefly, the maximum χ_ac_ at the optimal resonant frequency (*f*_res_), *f*_res_ corresponds to the maximum spin sensitivity. Therefore, Figure 2 plots the highest χ_ac_ as a function of MgO thickness. The resonance peak of origin 10 Hz represents to initial spin exchange coupling status. The maximum χ_ac_ value at 6 Å is 0.44, at 8 Å is 0.52, at 11 Å is 0.71, at 13 Å is 0.27, and at 15 Å is 0.3. These findings are known to follow from indirect interactions of magnetic moment. The indirect interactions of magnetic moment mean the spin exchange interaction between free CoFeB and pinned CoFeB layers, which indicate a strong moment interactions can induce a high magnetic susceptibility [24]. The susceptibility peaks relate to the exchange interaction between the two layers closely. The high χ_ac_ peaks are corresponding to high exchange coupling.

**Figure 3:**
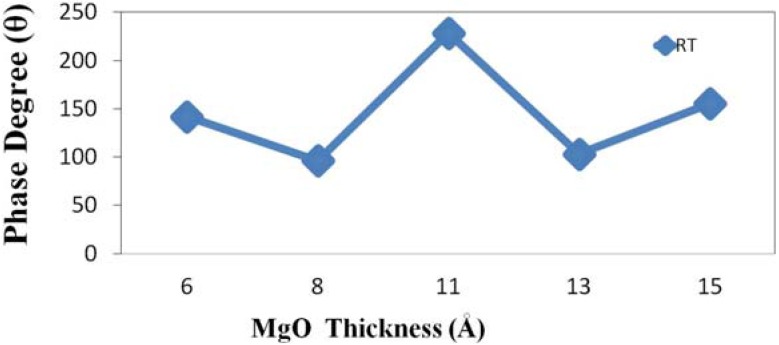
Variation of maximum χ_ac_ with maximum phase angle (θ).

The phase angle (θ) has the following physical meaning. When a magnetic material is in an external magnetic field, the magnetic dipole moment tends to lie in the direction of the interaction of the magnetic moment with the external field. When an external AC magnetic field is applied, and the AC frequency is not too high compared to microwave frequencies, the magnetic dipole moment oscillates. The frequency of the applied AC magnetic field equals the frequency of oscillation of magnetic dipole. However, the direction of instantaneous magnetic dipole is not the same as the direction of the applied magnetic field. The phase angle denotes the difference [29]. Figure 3 plots the corresponding maximum phase angle (θ_max_) between magnetic field and magnetization as a function of MgO thickness for maximal χ_ac_. The high χ_ac_ ensures high spin sensitivity to an increase in the phase angle. Restated, by increasing the phase angle improved the sensitivity to detect the behavior of an electron spin. In summary, the results concerning the phase angle are consistent with trend of χ_ac_.

Table 1 presents the important parameters of CoFeB/MgO/CoFeB MTJ. From this Table, an MgO thickness of 11 Å is the best of various MgO thicknesses from 6 to 15 Å. The maximum χ_ac_ of indirect exchange-coupling susceptibility of FM1 and FM2 is 0.7, corresponding to a resonant frequency of 10 Hz and a maximum phase angle of 228.5°. According to previous study, it indicates that susceptibility is associated with 1/f noise due to electron trap in the tunnel barrier [18]. It is related to electron traps and defects in the tunnel barrier but not related to the magnetization fluctuations, which suggests that the quality of the tunneling barrier is an important parameter in reducing low-frequency noise in magnetic tunnel junctions. According to the result, this CoFeB/MgO/CoFeB MTJ is suitable for components and low-frequency magnetic device applications [30].

**Table 1:** Maximum χ_ac_ value, maximum phase angle, and corresponding optimal resonance frequency for various MgO barrier thicknesses.

MgO (Å)	Maximum χ_ac_ (a.u.)	Maximun phase angle θ_max_ (degree)	Highest χ_ac_ corresponding optimal resonance frequency *f*_res_ (Hz)
6 Å	0.44	142.24	30 Hz
8 Å	0.52	96.44	30 Hz
11 Å	0.71	228.59	10 Hz
13 Å	0.27	103.47	10 Hz
15 Å	0.30	155.44	10 Hz

**Figure 4:**
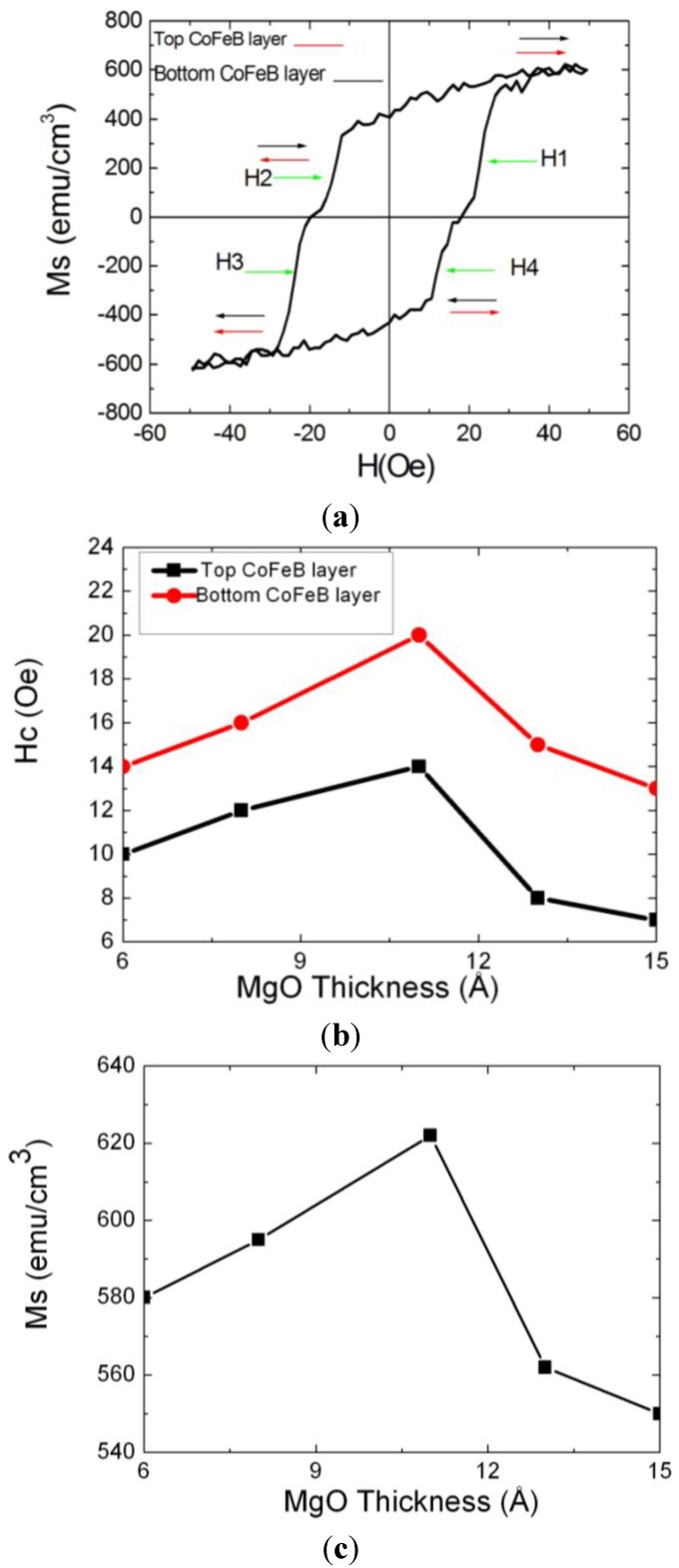
The essential magnetic properties of magnetic tunneling junction are (**a**) hysteresis loop of MTJ, (**b**) coercivity value, and (**c**) saturation magnetization.

Figure 4a shows the hysteresis loop of CoFeB(75 Å)/MgO(11 Å)/CoFeB(75 Å) MTJ. From this figure, the two-step characteristic of hysteresis loop is indicated that the spin rotated situation between two CoFeB layers at saturated magnetic magnetization by external field (H). Moreover, the H1, H2, H3, and H4 of Figure 4a indicate the coercive fields of the two CoFeB layers, respectively. The H_c_ value of free CoFeB layer denotes (H2 + H4)/2. The *H*_c_ value of pinned CoFeB layer is (H1 + H3)/2. The H_c_ value between two CoFeB layers initially increased at MgO thicknesses from 6 to 11 Å and decreased at MgO thicknesses from 11 to 15 Å. It can be reasonably concluded that high *H*_c_ value means high exchange-coupling χ_ac_ strength. High *H*_c_ strength requires a large external field to changing the spin arrangement. From Figure 4b, it suggests that the *H*_c_ of MTJ is varied by various MgO thicknesses. The result of Figure 4b is consistent with Figure 2. According to the results of Figure 2 and Figure 4b, they indicate that maximum χ_ac_ means a high magnetic exchange coupling between two CoFeB layers and induces a corresponding high *H*_c_. The saturation magnetization (*M*_s_) between two CoFeB layers is also shown the same trend to concave-down feature, which is shown in Figure 4c. From the result of Figure 4, it indicates that high M_s_ presents to high exchange-coupling χ_ac_ strength and high *H*_c_ value.

## 3. Experimental Section

A multilayer MTJ was sputtered on a glass substrate by a DC and RF magnetron sputtering system. The chamber pressure was typically under 1 × 10^−7^ Torr and the Ar-working chamber pressure was 5 × 10^−3^ Torr. The MTJ structure was glass/CoFeB(75 Å)/MgO(*d*)/CoFeB(75 Å) with *d* = 6, 8, 11, 13 and 15 Å. The atomic composition of the CoFeB alloy target was 40 atom % Co, 40 atom % Fe, and 20 atom % B. In the fabrication of MgO barrier, the initial metal magnesium (Mg) target was deposited on the bottom of ferromagnetic electrode, and then deposited a magnesium oxide (MgO) layer was formed by RF sputtering reaction in an oxidizing atmosphere using an Ar/O_2_ with a mixing ratio of 9:16. The in-plane low-frequency alternate-current magnetic susceptibility (χ_ac_) of MTJ was studied using an χ_ac_ analyzer (X_ac_Quan, MagQu, Taiwan). First, the referenced standard sample is calibrated by an χ_ac_ analyzer with an external field. Then, the measured sample is inserted to χ_ac_ analyzer. The driving frequency ranged from 10 to 25,000 Hz. The minimum frequency step is 20 Hz and that the frequency minimum is 10 Hz. The χ_ac_ is determined through the magnetization measurement. All measured samples had the same shape and size to eliminate the demagnetization factor. The χ_ac_ valve is arbitrary unit (a.u.), because the χ_ac_ result is corresponding to referenced standard sample. It is a comparative valve. Moreover, the in-plane coercivity (*H*_c_) and saturation magnetization (*M*_s_) of the two CoFeB layers were obtained using a superconducting quantum interference device (SQUID, Quantum Design MPMS5, San Diego, CA, USA).

## 4. Conclusions

The MgO barrier layer thickness in CoFeB/MgO/CoFeB MTJs was varied to measure low-frequency alternate-current magnetic susceptibility and magnetic properties. The highest χ_ac_ was obtained at a thickness of 11 Å, corresponding to an optimal resonance frequency of 10 Hz and a maximum phase angle of 228.5°. The best resonance frequencies are from 10 to 30 Hz, and this range of frequencies is useful for transformers, sensors, and magnetic read heads. Additionally, the important low-frequency alternate-current susceptibility and magnetic results demonstrate that the indirect spin exchange coupling of top CoFeB and bottom CoFeB layers in CoFeB/MgO/CoFeB oscillates.
